# Epidemiology and molecular identification of mycoplasma pneumoniae associated with respiratory infections in Zhejiang province, China, 2008‐2017

**DOI:** 10.1002/jcla.23460

**Published:** 2020-07-14

**Authors:** Qian Jiang, Fangfang Yang, Yumeng Peng, XiaoYan Dong, Yumei Ge

**Affiliations:** ^1^ Department of Clinical Laboratory Zhejiang Provincial People's Hospital People's Hospital of Hangzhou Medical College Hangzhou China; ^2^ Department of Laboratory Center Huamei Hospital University of Chinese Academy of Sciences (Ningbo No. 2 Hospital) Ningbo China; ^3^ School of Laboratory Medicine and Life Science Wenzhou Medical University Wenzhou China; ^4^ Bengbu Medical College Bengbu China; ^5^ The Second Medical College Zhejiang Chinese Medical University Hangzhou China

**Keywords:** acute respiratory infection, fluorescent PCR, Mycoplasma pneumoniae, Pharyngeal, swab

## Abstract

**Introduction:**

Mycoplasma pneumoniae is a common cause of respiratory infections in humans. The aim of this study was to investigate the infection of Mycoplasma pneumoniae (MP) in patients with acute respiratory tract infections in Zhejiang Province from 2008 to 2017, and to provide evidence for the early diagnosis and prevention of MP pneumonia.

**Methods:**

MP‐DNA was detected in nasopharyngeal swabs of patients with acute respiratory tract infection by real‐time fluorescent PCR (TaqMan probe). Statistical analysis and epidemiological investigation were carried out on the test results.

**Results:**

There were 10 296 patients with acute respiratory tract infection in Zhejiang Provincial People's Hospital from 2008 to 2017, including 4387 females and 5909 males. A total of 1251 MP‐DNA–positive patients were detected, with a total positive rate of 12.2% (1251/10296). Among 1251 patients with MP infection, 571 were female positive, with an average positive rate of 13.0% (571/4387), and 680 were male positive, with a positive rate of 11.5% (680/5909). From 2008 to 2017, the positive rates were 22.8% (33 cases), 20.9% (211 cases), 20.9% (350 cases), 5.5% (70 cases), 11.7% (136 cases), 15.2% (190 cases), 7.8% (94 cases), 5.9% (62 cases), 7.8% (56 cases), and 6.0% (49 cases), respectively. Of 1251 MP‐DNA–positive patients, 1243 (99.4%) were younger than 18 years old.

**Conclusions:**

Mycoplasma pneumoniae infection mainly occurs from late summer to autumn and in the age below 18 years, suggesting that early diagnosis and prevention of MP infection in adolescents should be emphasized.

## Graphical abstract

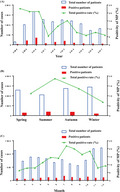

## 1 INTRODUCTION

Mycoplasma pneumonia (MP) is one of the most important pathogens of respiratory tract infection, and it is mainly transmitted by respiratory droplets.^1^, ^2^ It belongs to the class of flexible membranes. Mycoplasma is the smallest pathogenic microorganism between bacteria and viruses that can live independently. It has no cell wall and is naturally resistant to antibiotics acting on the cell wall.^3^ MP can cause acute, chronic respiratory infections, bronchitis, asthma, and other respiratory diseases.^4^, ^5^ It can also cause encephalitis, nephritis, myocarditis, and other extrapulmonary complications through blood dissemination or immune mechanism, especially in children's health.^6^, ^7^ In order to grasp the epidemic situation of MP infection in Zhejiang, to provide the basis for clinical diagnosis and treatment, and to facilitate the formulation of corresponding prevention and control strategies, the clinical results of MP infection in patients with acute respiratory tract infection who visited Zhejiang Provincial People's Hospital from 2008 to 2017 were analyzed.

## 2 MATERIALS AND METHODS

### 2.1 Specimen sources

Clinical data were collected from the information system of Zhejiang Provincial People's Hospital Inspection Center. This study was approved through the local ethics committee of Zhejiang Provincial People's Hospital. 10 296 nasopharyngeal secretion specimens were collected from patients in Zhejiang Provincial People's Hospital who were clinically diagnosed as respiratory tract infections between 2008 and 2017. Inclusion criteria included (a) specimens collected from patients with symptoms of upper respiratory tract infections and (b) written informed consent provided by the subject. Exclusion criteria included (a) specimens received in the laboratory in unsatisfactory containers or conditions, and (b) multiple sets of specimens from the same patient at different office visits. Among them, 4387 specimens were collected from female patients and 5909 specimens were collected from male patients. The throat swab specimens were placed in a sterile closed tube and stored at −20°C for testing. The 10 296 patients were divided into six age groups: <18 years old, 18‐29 years old, 30‐39 years old, 40‐49 years old, 50‐59 years old, and ≥60 years old.

### 2.2 The testing of MP

The samples were washed thoroughly by adding 1 mL sterile normal saline to the swab samples and shaking for 30 seconds. The samples were transferred to 1.5‐mL centrifuge tube by sterile suction tube. The samples were centrifuged for 10 minutes with 20 817*g* of Eppendorf freezing centrifuge. The supernatant was removed and the precipitate was shaken and mixed with 50 μL DNA extract solution, and then bathed at 100°C for 10 minutes. The supernatant was centrifuged for 5 minutes with 15 294*g*, and the supernatant was used as a template for PCR detection. Mycoplasma pneumoniae nucleic acid quantitative detection kit (PCR fluorescent probe method) was purchased from Da'an Co., Ltd. (National Instrument Accreditation 20153402024) and operated strictly according to the instructions. Lightcycler 480 was used for amplification reaction. The reaction procedure was preheated at 93 ℃ for 2 minutes, reacted at 93℃ for 5 seconds, and reacted at 57℃ for 45 seconds, with 40 cycles. The sensitivity of the kit was 1.0 × 10^4^ copies, and the linear range was 1.0 × 10^4^ ~ 1.0 × 10^8^ copies. If the amplification curve is S type and the CT value < 40, the sample is positive for Mycoplasma pneumoniae (+); if the CT is blank or the growth curve is not S type, it is negative for Mycoplasma pneumoniae (<500 copies).

### 2.3 Statistical analysis

Categorical variables were described with counts and percentages. The chi‐square test was used in comparisons of categorical variables. Statistical analyses were performed with SPSS 17.0 software. *P* value <.05 was considered significant.

## 3 RESULTS

### 3.1 Study Population

From 2008 to 2017, 10 296 patients with acute respiratory tract infections were treated in Zhejiang Provincial People's Hospital, including 4387 female patients and 5909 male patients. A total of 1251 MP‐DNA–positive patients were detected, with a total positive rate of 12.2% (1251/10296). Most of the patients who had MP infections (12.2%, 1251/10296) were children (12.3% vs 3.8%, *P* < .001) and female (13.0% vs 11.5%, *P* = .021). Of the infected cases, 143/1251 (11.4%) were treated as outpatients, and 1108/1251 (88.6%) patients were treated as inpatients (Table 1).

**TABLE 1:** Characteristic analysis on MP cases during 2008‐2017. Data were represented as n (%) in total cases and as n (positivity, %) in MP cases

Characteristic	Total	MP cases	χ^2^	*P* value
Age				
Children (<18 y)	10 087(98.0)	1243 (12.3)	13.843	<.001
Adults (≥18 y)	209(2.0)	8 (3.8)		
Gender				
Female	4387(42.6)	571 (13.0)	5.363	.021
Male	5909(57.4)	680 (11.5)		
Site of care				
Outpatients	813 (7.9)	143 (17.6)	24.462	<.001
Inpatients	9483(92.1)	1108 (11.7)		
Total	10 296	1251 (12.2)		

Note

*P* value: Statistical analyses were performed within MP cases.

### 3.2 Annual, quarter, and monthly cases from 2008 to 2017

**FIGURE 1:**
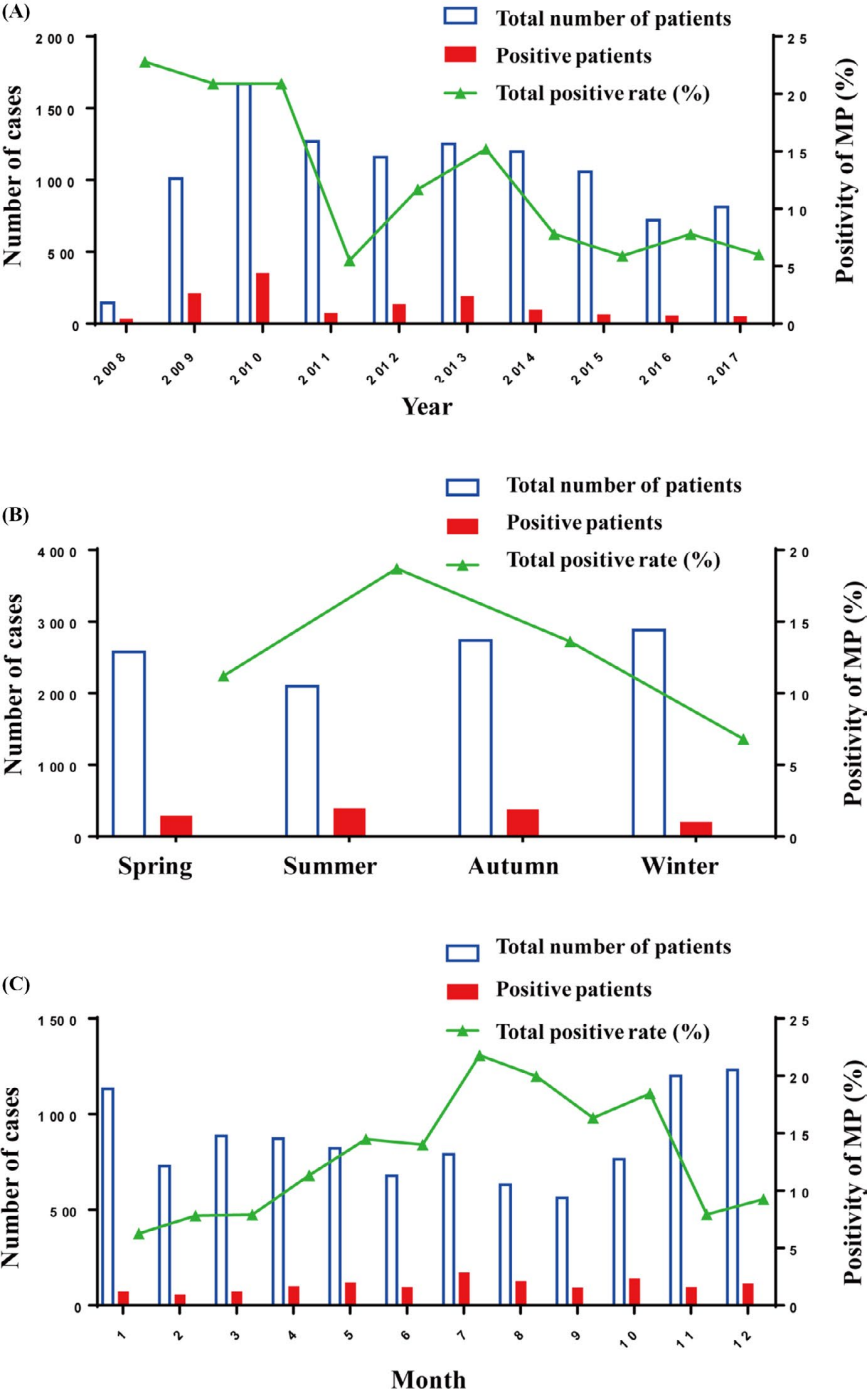
Annual, quarter, and monthly cases and positivity of MP from 2008 to 2017

From 2008 to 2017, the positive rates were 22.8% (33 cases), 20.9% (211 cases), 20.9% (350 cases), 5.5% (70 cases), 11.7% (136 cases), 15.2% (190 cases), 7.8% (94 cases), 5.9% (62 cases), 7.8% (56 cases), and 6.0% (49 cases), respectively (Figure 1A). The highest positive rate of MP‐DNA was 22.8% in 2008. In recent years, the positive rate of MP‐DNA was decreasing year by year. The positive rate of MP in spring was 11.2% (288 cases), summer was 18.7% (393 cases), autumn was 13.6% (373 cases), and winter was 6.8% (197 cases) (Figure 1B). The monthly cases for the total study period are shown in Figure 1C. There were four peaks with the positive rate of MP over 16%, increased from late summer to autumn, and lasted for 4 to 5 months. The lowest positive rate of MP was seen as 6.27% in January.

### 3.3 Analysis of 10 296 MP patients from 2008 to 2017 in different gender groups

Among 1251 patients with MP infection, 571 were female positive, with an average positive rate of 13.0% (571/4387), and 680 were male positive, with a positive rate of 11.5% (680/5909). There are more male patients than female patients, but there is no significant difference between the positive rate of MP‐DNA in male patients and the positive rate of MP‐DNA in female patients **(**Table. 2 **)**.

**TABLE 2:** Analysis of 10 296 MP patients from 2008 to 2017 in different gender groups. Data were represented as n (%) in total cases and as n (positivity, %) in MP cases

Group	Total		MP cases	
	Male patients	Female patients	Male positive patients	Female positive patients
Total	5909 (57.4)	4387 (42.6)	680 (11.5)	571 (13.0)
2008	77 (53.1)	68 (46.9)	15 (19.5)	18 (26.5)
2009	570 (56.4)	440 (43.6)	101 (17.7)	110 (25.0)
2010	923 (55.2)	748 (44.8)	189 (20.5)	161 (21.5)
2011	782 (61.6)	487 (38.4)	47 (6.0)	23 (4.7)
2012	688 (59.4)	471 (40.6)	74 (10.8)	62 (13.2)
2013	744 (59.4)	509 (40.6)	108 (14.5)	82 (16.1)
2014	710 (59.2)	489 (40.8)	57 (8.0)	37 (7.6)
2015	574 (54.3)	484 (45.7)	32 (5.6)	30 (6.2)
2016	395 (54.9)	325 (45.1)	27 (6.8)	29 (8.9)
2017	446 (54.9)	366 (45.1)	30 (6.7)	19 (5.2)
*P*		.001		.154
χ^2^		27.592		13.206

### 3.4 Analyses on MP cases by age groups

10 296 patients with acute respiratory tract infection were divided into six age groups: <18 years old (10 087 cases), 18‐29 years old (36 cases), 30‐39 years old (29 cases), 40‐49 years old (24 cases), 50‐59 years old (18 cases), and ≥60 years old (102 cases). The number of positive patients in each age group was < 18 years old (1243 cases), 18‐29 years old (1 case), 30‐39 years old (3 cases), 40‐49 years old (1 case), 50‐59 years old (0 cases), and ≥ 60 years old (3 cases). Among them, the number of patients with acute respiratory tract infection was the highest and the incidence of M. pneumoniae infection was the highest in the age group < 18 years old. The results are shown in Table 3.

**TABLE 3:** Analyses on MP cases by age groups

Group	Age groups (years)	Total	Years									
			2008	2009	2010	2011	2012	2013	2014	2015	2016	2017
Patients (n)	<18	10 087	140	992	1650	1259	1142	1233	1171	1022	700	778
	18‐29	36	0	2	3	2	3	5	4	7	4	6
	30‐39	29	0	2	2	1	4	2	5	2	6	5
	40‐49	24	0	0	1	1	1	5	3	4	2	7
	50‐59	18	1	0	1	2	4	1	5	2	1	1
	≥60	102	4	14	14	4	5	7	11	21	7	15
Positive patients	<18	1243	33	211	348	70	136	189	92	61	54	49
	18‐29	1	0	0	1	0	0	0	0	0	0	0
	30‐39	3	0	0	0	0	0	0	1	0	2	0
	40‐49	1	0	0	0	0	0	1	0	0	0	0
	50‐59	0	0	0	0	0	0	0	0	0	0	0
	≥60	3	0	0	1	0	0	0	1	1	0	0

## 4 DISCUSSION

Mycoplasma pneumoniae mainly transmitted by droplets with a latency of 2‐3 weeks. After infection, it can adhere to the surface of respiratory tract cells, then extend microtubules into cells to absorb nutrients and damage cell membranes, and then release nuclease, hydrogen peroxide, and other metabolic products to cause cell lysis and swelling and necrosis of epithelial cells.^8^, ^9^, ^10^ Mycoplasma pneumoniae infection can cause a variety of respiratory diseases and also can cause a variety of extrapulmonary complications after blood dissemination.^6^, ^11^ The pathological changes in mycoplasma pneumonia were mainly interstitial pneumonia, and the incidence of mycoplasma pneumonia was the highest among adolescents. The clinical manifestations and chest X‐ray examination of mycoplasma pneumonia are not characteristic.^11^, ^12^, ^13^ Diagnosis cannot be made solely by clinical manifestations and chest X‐ray examination. It is easy to cause clinical missed diagnosis. To make a definite diagnosis, pathogen detection is needed.

Mycoplasma pneumoniae lacks cell wall and is highly pleomorphic and can filter bacteria.^3^, ^14^ Therefore, penicillin, cephalosporin, and other antibiotics acting on bacterial cell walls have poor therapeutic effect. Mycoplasma pneumoniae is generally sensitive to macrolides and tetracyclines, so rapid detection of MP is of great value for early clinical diagnosis and antibiotic treatment.

At present, the detection methods for MP mainly include MP separation culture method, immune serum method, and PCR method.^15^, ^16^ Although isolation and culture of MP are decisive for diagnosis and differential diagnosis, its operation is complicated, time‐consuming, and low in detection rate, and often cannot meet clinical needs.^17^ Serological methods are often used in clinical practice. However, there are fewer anti–MP‐IgM antibodies produced in the early stage of infection, and it usually takes about 1 week to be detected by serology, which delays the optimal treatment timing.^18^ And for patients with immune system development or immune function defects, the detection rate of anti–MP‐IgM antibodies is low. Therefore, the detection of MP‐DNA by real‐time fluorescent PCR has become more and more popular.

The positive detection rate of MP varies greatly among different countries and regions, different populations and ages, different years, and seasons,^1^, ^19^, ^20^ in order to grasp the epidemic situation of MP infection in Zhejiang, provide evidence for clinical diagnosis and treatment of antibiotics, and formulate prevention and treatment strategies. This paper summarizes the MP infection of patients with acute respiratory infection in this area. From 2008 to 2017, there were 10 296 patients with acute respiratory infections in the People's Hospital of Zhejiang Province, including 4387 females and 5909 males. A total of 1251 MP‐DNA–positive patients were detected, with a total positive rate of 12.2%. The previously reported MP infection rates varied from 8.7% to 20.7% in different regions.^21^, ^22^, ^23^ The infection rate of MP in this area is at a low level, which is related to the high medical level in this area. Judging from the degree of seasonal prevalence, this result shows that the positive cases of MP are mainly concentrated in summer, of which more positive rates are shown from July to October. In areas with moderate temperatures, outbreaks of MP infections are mainly concentrated in summer or early autumn.^24^ Therefore, this area should pay more attention to the clinical concern of MP incidence from July to October, rather than the cold season with high incidence of respiratory diseases as we used to think. From 2008 to 2017, the positive rates were 22.8%, 20.9%, 20.9%, 5.5%, 11.7%, 15.2%, 7.8%, 5.9%, 7.8%, and 6.0%, respectively. The infection rate of MP showed a general downward trend. Although there are occasional fluctuations, it means that the infection control of MP in this area has improved year by year. Among 1251 patients with MP infection, 571 were female positive, with a positive rate of 13.0% (571/4387), and 680 were male positive, with a positive rate of 11.5% (680/5909). Of 1251 MP‐DNA–positive patients, 1243 (99.4%) were younger than 18 years old. It indicates that MP infection is mainly concentrated in the age group below 18, suggesting that clinical attention should be paid to the early diagnosis and prevention of MP infection in adolescents.

## 5 CONCLUSIONS

The rapid detection of MP by real‐time PCR has important clinical value for rational selection of antibiotics and early diagnosis. Clinical attention should be paid to MP‐DNA testing in patients with acute respiratory infections from late summer to autumn and in the age of 18 or younger. For patients with respiratory infections that are poorly treated with antibiotics acting on bacterial cell walls, the possibility of MP infection should be considered.

## ETHICAL APPROVAL

This study was approved by the local ethics committee of Zhejiang Provincial People's Hospital. The study has been performed in accordance with the ethical standards laid down in the 1964 Declaration of Helsinki.

## COMPLIANCE WITH ETHICAL STANDARDS

The authors declare that they have compliance with ethical standards.
